# Chromatin Evolution-Key Innovations Underpinning Morphological Complexity

**DOI:** 10.3389/fpls.2019.00454

**Published:** 2019-04-12

**Authors:** Mohsen Hajheidari, Csaba Koncz, Marcel Bucher

**Affiliations:** ^1^Botanical Institute, Cologne Biocenter, Cluster of Excellence on Plant Sciences, University of Cologne, Cologne, Germany; ^2^Department of Plant Developmental Biology, Max Planck Institute for Plant Breeding Research, Cologne, Germany; ^3^Biological Research Center, Institute of Plant Biology, Hungarian Academy of Sciences, Szeged, Hungary

**Keywords:** gene duplication, evolution, chromatin, transcriptional regulation, morphological complexity, microbiota, symbiosis

## Abstract

The history of life consists of a series of major evolutionary transitions, including emergence and radiation of complex multicellular eukaryotes from unicellular ancestors. The cells of multicellular organisms, with few exceptions, contain the same genome, however, their organs are composed of a variety of cell types that differ in both structure and function. This variation is largely due to the transcriptional activity of different sets of genes in different cell types. This indicates that complex transcriptional regulation played a key role in the evolution of complexity in eukaryotes. In this review, we summarize how gene duplication and subsequent evolutionary innovations, including the structural evolution of nucleosomes and chromatin-related factors, contributed to the complexity of the transcriptional system and provided a basis for morphological diversity.

## Introduction

**FIGURE 1:**
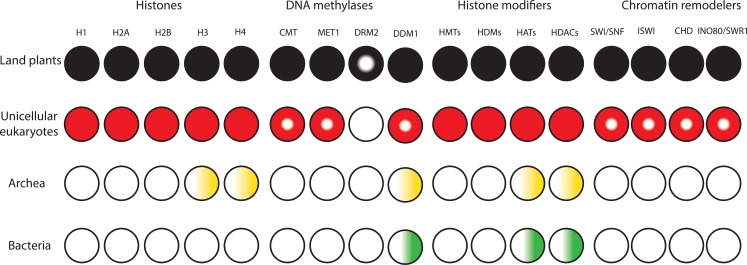
Diversification and expansion of histones, chromatin remodelers and modifiers in the domains of life. Filled circles denote presence of orthologs in all lineages. Semi-filled circles indicate factors present in part of the lineage. Gradients indicate presence of homologs. White circles indicate the lack of homologs.

Early organisms on Earth were microscopic, and for the first 2500 million years (Myr), living organisms rarely achieved a complexity higher than two or three cell types (Carroll, 2001). Around 500 Myr ago from the mid-Cambrian to early Ordovician, land plants that are a major focus of this review likely evolved from a lineage of unicellular eukaryotes in charophyte green algae (Stebbins and Hill, 1980; Kenrick and Crane, 1997; Harholt et al., 2016; Del-Bem, 2018; Morris et al., 2018). With the evolution of land plants, these more complex organisms colonized the Earth and transformed the biosphere providing habitable environments for terrestrial organisms by supplying sufficient oxygen and nutrients (Hori et al., 2014). Recent evolutionary analyses indicate that the cell wall, symbiotic signaling pathways, the RPB1 heptapeptide repeats, hormonal biosynthesis or signaling pathways, and desiccation and UV radiation tolerance evolved in charophyte green algae prior to land plants (Stebbins and Hill, 1980; Hajheidari et al., 2013; Hori et al., 2014; Yang and Stiller, 2014; Delaux et al., 2015; Ju et al., 2015; Harholt et al., 2016; Del-Bem, 2018). This demonstrates that charophyte green algae were preadapted to cope with harsh terrestrial environments. The greater complexity of unicellular eukaryotes and the evolution and diversification of land plants could not be possible without the existence of a high level of cellular complexity and elaborate mechanisms for gene regulation in unicellular eukaryotic ancestors (Figure 1).

Eukaryotes have a high degree of cellular complexity. The genomes of most eukaryotes are larger than those of prokaryotes, however, in eukaryotes, in contrast to prokaryotes, genome size does not show a good correlation with gene number (Valentine, 1978; Gregory, 2005). Furthermore, an increase in genome size or gene number is not a good criterion for developmental and morphological complexity. For example, the genome of the bryophyte *Physcomitrella patens* is about 480 MB and possesses approximately 35,938 genes, while *Arabidopsis thaliana*, with much higher morphological complexity, has a smaller genome (∼135 MB) containing about 27,235 genes (Rensing et al., 2008). To understand the evolution and diversification of morphological complexity two questions should be addressed. First, which factors were the major genetic resources underpinning morphological complexity? And secondly, how does morphological diversity evolve? We know that all cells of a complex multicellular organism contain the same genome, however, their organs are composed of a variety of cell types that differ dramatically in both structure and function. The distinctiveness of a given cell type is determined by controlled transcriptional activity of distinct sets of genes in a cell lineage. Complexity is a term with different definitions (Carroll, 2001). However, the number of cell types is broadly considered an indicator for morphological/organismal complexity (Carroll, 2001; Levine and Tjian, 2003; Chen et al., 2014). This suggests that complex transcriptional regulation plays a key role in the evolution of complexity in eukaryotes. This is in agreement with a higher proportion of transcription factors in more complex organisms with high evolutionary distances within each kingdom (Table 1). Moreover, the rate of expansion of transcriptional regulators is faster than linearly for every gene added to the genome (Levine and Tjian, 2003; Charoensawan et al., 2010a; Rensing, 2014). However, in many cases evolution is not necessarily accompanied by higher morphological complexity or with an increased number of transcriptional regulators (Wolf and Koonin, 2013).

**Table 1:** Organismal/morphological complexity correlates with the proportion of transcriptional regulators within each kingdom when the evolutionary distance between organisms is high.

Kingdom	Species	No. of cell types	Genome size	No. of genes	No. of TFs	Proportion of TFs
Metazoa	*Homo sapiens*	264.5	∼ 3.3 GB	∼ 22997	∼ 1508	0.0656
	*Mus musculus*	130.5	∼ 2.7 GB	∼ 23873	∼ 1426	0.0597
	*Tetraodon nigroviridis*	119.5	∼ 390 MB	∼ 27991	∼ 1362	0.0487
	*Drosophila melanogaster*	59	∼ 175 MB	∼ 14141	∼ 601	0.0425
	*Caenorhabditis elegans*	28.5	∼ 100 MB	∼ 20140	∼ 698	0.0347
	*Nematostella vectensis*	22	∼ 450 MB	∼ 27273	∼ 701	0.0257
	*Trichoplax adhaerens*	4	∼ 50 MB	∼ 11520	∼ 233	0.0202
Viridiplantae	*Zea mays*	100	∼ 2.5 GB	∼ 45796	∼ 2689	0.0587
	*Arabidopsis thaliana*	27.25	∼ 135 MB	∼ 27235	∼ 1356	0.0498
	*Selaginella moellendorffii*	25	∼ 100 MB	∼ 22273	∼ 665	0.0299
	*Physcomitrella patens*	21	∼ 480 MB	∼ 35938	∼ 823	0.0229
	*Marchantia Polymorpha*	NA^∗^	∼ 225 MB	∼ 32718	∼ 586	0.0179
	*Klebsormidium nitens*	1	∼ 117 MB	∼ 16215	∼ 273	0.0168
	*Chlamydomonas reinhardtii*	1	∼ 107 MB	∼ 15256	∼ 213	0.0140
	*Chlorella* sp. NC64A	1	∼ 46.2 MB	∼ 9791	∼ 131	0.0134


The arrangement of organisms within each kingdom is based on organismal complexity. Proportion of transcriptional factors (TFs) represents the ratio of number of TFs to number of genes. Data were mostly obtained from transcription factor prediction database, only the longest transcript per gene was included in this study (http://www.transcriptionfactor.org) (Valentine et al., 1994; Bell and Mooers, 1997; McCarthy and Enquist, 2005; Vogel and Chothia, 2006; Charoensawan et al., 2010b; Burdo et al., 2014; Chen et al., 2014; Hori et al., 2014; Matsumoto et al., 2016; Jiao et al., 2017). ^∗^NA, not available.

## Gene Duplication - a Major Driver in the Evolution of Morphological Complexity

Genomic studies have revealed notable increases in the number of genes, intergenic regions, intragenic regions (introns), and transposons from prokaryotes to multicellular eukaryotes. Whole-genome and small-scale duplications are known as essential sources for the evolution of functional novelty and morphological complexity (Ohno, 1970; Lynch and Conery, 2003; Gregory, 2005; Bratlie et al., 2010). Increases in organismal complexity are repeatedly coupled to short-term large-scale increases in gene number in the history of eukaryotes (Ohno, 1970; Gu et al., 2002; McLysaght et al., 2002; Maere et al., 2005; Vanneste et al., 2014). For example, eukaryotic RNA polymerases (PolI, PolII, and PolIII) evolved due to massive gene duplications during the transition from an archaeum to a fully fledged eukaryote (Koonin, 2015). Whole-genome duplications in plants normally lead to genomic instability, alteration of gene expression and cell division abnormalities (Comai, 2005). On the other hand, the genomic plasticity of polyploids is higher than diploids and this may lead to increased tolerance of polyploidy in a broader range of environmental conditions. Recent studies suggest that challenging environmental conditions may positively enhance short-term polyploid establishment and survival (for a detailed review see, Van de Peer et al., 2017). After genome duplication, duplicated genes can have different evolutionary fates. Duplicated genes predominantly become pseudogenes/silent due to non-adaptive accumulation of deleterious mutations (non-functionalization) within a few Myr (Lynch and Conery, 2003; Maere et al., 2005). In an evolutionary study in rodents, it was shown that one copy of duplicates, which is usually the novel daughter copy, experiences a fivefold higher divergence rate within 4–12 Myr after duplication. Subsequently, the divergence rate decreases and after 40.5 Myr returns to preduplication levels (Pegueroles et al., 2013). A subset of duplicates may stay active by different mechanisms. For example, an increase in the expression of duplicates can be beneficial (gene dosage) or both duplicates can be essential to keep the ancestral function (subfunctionalization). In addition, duplicates can be important to maintain the stoichiometric balance (gene balance) or to prevent interference between the products of paralogs (paralog interference). Duplication can also lead to the evolution of novel functions. Neofunctionalization arises after gene duplication resulting in one gene copy keeping the ancestral function and the second copy becoming fixed by positive selection. In addition, functional novelty can also arise due to escape from adaptive conflict (EAC). In this case, the evolution of a novel function in the ancestral copy before duplication has reduced the ability of the gene to carry out the original function and after duplication each copy can freely optimize the ancestral or the novel function (Ohno, 1970; Lynch, 2000; He and Zhang, 2005; Conant and Wolfe, 2008; Des Marais and Rausher, 2008). It has been shown that the decay rates of paralogs derived from small-scale duplications are considerably higher than those derived from large-scale duplications (Maere et al., 2005; Freeling, 2009). Furthermore, after whole genome duplication the retention rate of different genes is not similar. For example, genes that are involved in transcriptional regulation, signal transduction, and development have a higher retention rate than other functional categories (Blanc, 2004; Seoighe and Gehring, 2004; Maere et al., 2005). On the other hand following a large-scale duplication and emergence of polyploid organisms, most of the duplicates are deleted or non-functionalized over time and genome size reduction is accompanied by extensive genome reorganization. This process is called diploidization and leads to the conversion of polyploids to diploids over a period of several Myr and species that emerge by diploidization following polyploidization are called palaeopolyploids. All extant angiosperms are indeed palaeopolyploid (Olsen and Wendel, 2013; Dodsworth et al., 2016). It is also important to consider that whole-genome duplication in animals in general is less common than in plants (Hallinan and Lindberg, 2011; Nossa et al., 2014; Clarke et al., 2015; Schwager et al., 2017; Li et al., 2018).

## Alteration of Gene Expression Patterns and Morphological Complexity

Pioneering studies in molecular evolutionary biology revealed that there is relatively little protein divergence among mammalians such as chimps and humans, although their phenotype and behavior are very different (Britten and Davidson, 1971; Wilson et al., 1974; King and Wilson, 1975). These studies led to the proposal that the evolution of complexity occurred more by altering gene regulation than by changing protein sequences. In agreement with this proposal, later studies showed that many homologous proteins, despite long term (∼ 500 Myr) independent evolution in different lineages, are often functionally equivalent (Grens et al., 1995; Halder et al., 1995). Furthermore, vital roles are attributed to conserved protein sequences and their mutations are deleterious or lead to pleiotropic effects and are thus under purifying selection (Grens et al., 1995; Halder et al., 1995; Hoekstra and Coyne, 2007). However, alteration of their expression level or pattern is usually non-deleterious and this is mostly due to the modular nature of *cis-*regulatory elements (Hoekstra and Coyne, 2007).

The morphological complexity of multicellular organisms relies on spatio-temporal patterns of developmentally important regulatory factors (Spitz and Furlong, 2012). The precise expression patterns of master developmental regulators are mostly governed by enhancers/*cis-*regulatory modules that integrate signaling and tissue-specific inputs to specify times and locations of gene expression (Shen et al., 2012). Enhancers are short DNA sequences that contain multiple sites for sequence-specific transcription factors (Shlyueva et al., 2014). In prokaryotes, enhancer-dependent gene regulation is less common and the regulatory regions of prokaryotes and unicellular eukaryotes are usually composed of short sequences in the vicinity of the core promoter (Gralla, 1996; Wyrick and Young, 2002). However, enhancers in multicellular eukaryotes are scattered across the genome and found upstream and downstream of genes. The birth of enhancers is mediated by various mechanisms during evolution. Duplication and rapid/subsequent diversification of enhancers is an important source for the genesis of new enhancers (Goode et al., 2011; Vlad et al., 2014). New enhancer sequences can emerge from non-regulatory sequences or older enhancer elements via random genetic drift or adaptive selection (Frankel et al., 2011; Rebeiz et al., 2011; Duque and Sinha, 2015; Villar et al., 2015). Transposable elements (TEs) are also important material for tinkering with eukaryotic transcriptional regulatory systems (Jordan et al., 2003; Cao et al., 2016). Enhancers can control genes that are located far away; therefore, one gene can be regulated by multiple distal and close enhancers with different spatiotemporal activities. Furthermore, one enhancer may regulate the activity of multiple genes. These features facilitate a vast combinatorial complexity of transcriptional regulation with a relatively limited set of genes (Long et al., 2016).

It is important to consider that alteration in heritable gene expression patterns is due either to diversification of *cis*-regulatory elements or *trans*-regulatory factors (transcription regulators and non-coding RNAs). Recent studies have quantified the relative contribution of *cis*- and *trans*-regulatory factors to the evolution of gene expression, which as shown above is a key player in the evolution of morphological complexity. These studies suggested that *trans*-regulatory factors have a higher contribution to gene expression alteration than *cis*-regulatory factors within a given species. However, as sequence divergence or evolutionary distance increase, *cis*-regulatory differences become the dominant contributor in gene expression alteration. This relative contribution of *cis*-regulatory elements and *trans*-regulatory factors in the regulation of gene expression varies amongst taxa (Metzger et al., 2017; Osada et al., 2017).

Many studies have shown evolutionary changes through diversification of regulatory elements or protein-coding sequences (Stern, 1998; Arnaud et al., 2011; Vlad et al., 2014; Kusters et al., 2015; Sicard et al., 2016; Vuolo et al., 2016; Jiang and Rausher, 2018). Reduced complexity (RCO) evolution is an interesting example in plants that shows how gene duplication and subsequent diversification in regulatory elements and coding sequences played a key role in the evolution of morphological diversity within the Brassicaceae family. Vlad et al. (2014) discovered that a tandem duplication of the *LATE MERISTEM IDENTITY 1 (LMI1)* gene has given rise to two new copies in Cardamine. One of the copies has become a pseudogene owing to accumulation of deleterious mutations, whereas another copy located immediately downstream of the *LMI1*gene locus is active. *LMI1* is expressed in the margins of leaflet, stipules, and flowers. In contrast to *LMI1*, the novel active copy *RCO* is essential for the formation of the complex leaves in *C. hirsuta*. It is expressed at the base of the leaflet and promotes leaflet formation through local growth repression. *RCO* was lost in the lineage that gave rise to *A. thaliana* leading to simplification of the leaves in this species. When the *RCO* promoter drives the expression of the *LMI1* gene at the base of leaflets, the *LMI1* gene acts similar to *RCO* and represses the growth at the flank of developing leaflets. This demonstrated that neofunctionalization has occurred due to diversification of regulatory elements. Later studies uncovered that indeed RCO enhancer evolution likely coevolved with a single amino acid change. This change led to the reduction of RCO protein stability, which is required for minimizing the pleiotropic effects of the RCO enhancer (Vuolo et al., 2016). The evolution of domesticated maize (*Zea mays* ssp. *mays*) from its wild relative teosinte (*Z. mays*, ssp. *Parviglumis*) is also an excellent example of morphological evolution through directional selection during domestication. Since the crop plant maize and teosinte are morphologically very different, taxonomists once placed them in separate genera (Doebley et al., 1997). However, later studies demonstrated that these plants are close relatives and expression alteration of a few transcription factors led to great morphological divergence and played a substantial role in the emergence of cultivated maize from teosinte. Diversification of regulatory elements of *teosinte branch1* (*tb1*) and *barren stalk1* (*ba1*), which encode bHLH transcription factors, had a great impact on positioning of the male inflorescence and conversion of lateral branches of teosinte into the maize ear (Doebley et al., 1997; Gallavotti et al., 2004; Clark et al., 2006). In teosinte, kernels are tightly sealed in a stony casing, while the kernels of crop maize are naked and could readily be consumed by animals or humans. Surprisingly, just a single amino acid change in the SBP-box transcription factor *teosinte glume architecture1* (*tga1*) was the cause of the liberation of kernels from the hardened cupulate fruitcases (Wang et al., 2005, 2015).

## The Structural Evolution of the Nucleosome as a Prerequisite Step for Morphological Complexity

To the best of our knowledge, all domains of life rely on DNA to store and inherit genetic information. Factors that alter the conformation of DNA to make it fit inside the cell/nucleus are present in all kingdoms of life and have the potential to influence transcription. Bacteria lack histones and contain nucleoid-associated proteins (NAPs) that are major DNA-binding factors facilitating chromosomal domain formation and organization (Figure 1; Luijsterburg et al., 2008). In bacterial cells, there is no inherent barrier for RNA polymerases to gain access to the DNA (Struhl, 1999; Dillon and Dorman, 2010). Archaeal cells also have circular DNA, as in bacteria. The phylum Crenarchaeota in the archaea domain generally lack histone proteins and their chromosome organization relies on Alba proteins, which are NAPs. However, the phylum Euryarchaeota in archaea mainly contain histone proteins that lack flexible tails at their N-terminus (Williams and Embley, 2014; Peeters et al., 2015). *Methanopyrus kandleri* and *Halobacterium* NRC1 in Euryarchaeota contain unusual “doublet histones” that have evolved through an end-to-end duplication of the histone fold. The ancestral gene encoding a doublet histone was split and diverged into H3 and H4 to form H3–H4 tetramers. H2A and H2B likely evolved later through a second specialization of a doublet as well (Ng et al., 2000; Malik and Henikoff, 2003). Eukaryotic histones are derived from a common ancestor shared with Archaea. Archaeal chromatin-like structure is apparently important for DNA protection from thermal denaturation (Reeve, 2003; Sandman and Reeve, 2005). Eukaryotic cells contain very stable, compact, and at the same time very dynamic chromatin. Nucleosomes are the fundamental units of chromatin that consist of ∼147 base pairs of DNA wrapped around a core of eight histone proteins comprising two copies of histone H3, H4, H2A, and H2B. The tails of core histones protrude from the nucleosome core particle and many residues in these tails can be post-translationally modified, influencing all DNA-based processes, including transcription (Venkatesh and Workman, 2015). Chromatin also contains linker DNAs (∼10–90 bp) that connect nucleosomes and interact with histone H1 (Han and Grunstein, 1988; Szerlong and Hansen, 2011; Zhou et al., 2013). In higher eukaryotes, H1 histones have three domains, a highly conserved central globular domain, an unstructured short N-terminal domain, and a long basic C-terminal domain (Ramakrishnan et al., 1993). Linker histone-like proteins are found in eubacteria, which are likely the provenance of H1 histones (Kasinsky et al., 2001). These proteins are similar to the C-terminal domain of H1 histones in higher eukaryotes, however, they have no globular domain. Linker histones are diverse and perform various roles in processes such as chromatin organization, genome stabilization, transcriptional regulation, and embryogenesis (Hergeth and Schneider, 2015; Kotliński et al., 2016; Bayona-Feliu et al., 2017). In contrast to prokaryotes, the compact structure of chromatin in eukaryotes generated an inherent barrier for DNA-based processes. This was one of the key prerequisite steps in the evolutionary trajectory of complex multicellular organisms.

## Evolution of Chromatin Remodelers and Modifiers

The compact structure of chromatin in eukaryotes prevents free access of transcription factors to *cis*-regulatory DNA elements. In addition to transcription factors, proteins involved in replication and repair must be able to access DNA. To tackle this barrier, it was necessary for early eukaryotes to evolve and expand classes of chromatin modifiers and remodelers to facilitate access to DNA (Figure 1). Due to the possession of mitochondria, Eukaryotes had more available energy to encode a higher level of proteins. This together with genome expansion likely generated evolutionary pressure for co-evolution of high density chromatin packaging and chromatin-modifying factors in early eukaryotes (Flaus et al., 2006; Lane and Martin, 2010; Garg and Martin, 2016; Koster et al., 2015; Martin and Sousa, 2016). Chromatin modifiers and remodelers further expanded and diversified in eukaryotes. This led to the establishment of distinct classes of chromatin-modifying factors with unique functional complexes that facilitate binding of transcription factors to *cis*-regulatory DNA elements in a cell-type-specific manner in higher eukaryotes (Gentry and Hennig, 2014; Sarnowska et al., 2016; Zhou et al., 2016). The major chromatin-modifying factors are DNA methyltransferases (DNMTs), histone deacetylases (HDACs), histone acetyltransferases (HATs), histone methyltransferases (HMTs), histone demethylases (HDMs), and chromatin remodelers.

### DNA Methyltransferases

In prokaryotes, as a part of the restriction-modification (RM) systems DNA methylases cooperate with restriction enzymes to protect the genome against foreign DNA. Prokaryotic DNA methylases evolved from ancient RNA-modifying enzymes and are the provenance of eukaryotic DNA methylases. In eukaryotes, multiple independent duplications, losses, and divergences led to the emergence of distinct types of DNA methylases, which are involved in a range of activities, including gene and transposon silencing, imprinting, transcriptional activation, and post-transcriptional regulation (Law and Jacobsen, 2010; Blow et al., 2016; Lyko, 2018). In Arabidopsis, *de novo* cytosine methylation is catalyzed by DOMAINS REARRANGED METHYLTRANSFERASE2 (DRM2) and the DNA methylation pattern is maintained by METHYLTRANSFERASE 1 (MET1) and CHROMOMETHYLASE 3 (CMT3), as well as DRM2. Interestingly, DNA methylation could create a basis for morphological diversity by regulating DNA binding affinity of transcription factors. For example, epigenetic mutation of the *Lcyc* gene inhibits its expression and modifies the symmetry of the flowers from bilateral to radial in *Linaria vulgaris* (Cubas et al., 1999). DNA hyper-methylation in the promoter region of a SBP-box transcription factor, COLORLESS NON-RIPENING (Cnr), leads to colorless and abnormal ripening of fruits in tomato without changes in nucleotide sequence (Manning et al., 2006). DNA methylation in eukaryotes can also be guided by non-coding RNAs. Small RNA-directed DNA methylation (RdDM) pathways play a key role in maintenance of genome stability and developmental regulation (Castel and Martienssen, 2013; Matzke and Mosher, 2014). The canonical RdDM model suggests that the target loci are transcribed by Pol IV and the primary transcripts are converted to dsRNAs by RDR2. These dsRNAs are processed into mature 24nt repeat-associated siRNA (ra-siRNA) by DCL3, methylated by HEN1, and loaded into RISC-like RITS (RNA-induced transcriptional silencing) complexes containing AGO4 and Pol V, which scan the genomic DNA to drive DNA methylation at target loci carrying complementary sequences (Cao et al., 2003; Zilberman et al., 2003; Wierzbicki et al., 2008; Law and Jacobsen, 2010).

The MORC ATPase family is an evolutionary conserved protein family that is prevalent in both prokaryotes and eukaryotes (Iyer et al., 2008). However, in eukaryotes, especially in the plant kingdom it greatly expanded through gene duplication (Dong et al., 2018). Using contextual information, Iyer et al. (2008) suggested that MORC proteins may play a substantial role in the bacterial RM system. MORC proteins are required for meiotic division in animals and pathogen-associated molecular pattern (PAMP)-triggered immunity in plants (Watson et al., 1998; Kang et al., 2012; Liu et al., 2016; Dong et al., 2018). The Arabidopsis genome contains seven *MORC* genes (*AtMORC1-7*). It has been demonstrated that MORC1, MORC2, and MORC6 are involved in gene silencing and transposon suppression without changing genome-wide DNA methylation patterns (Moissiard et al., 2012, 2014; Bordiya et al., 2016). However, MORC-mediated transcriptional silencing depends, at least in part, on the interaction with the RdDM components (Lorković et al., 2012; Brabbs et al., 2013; Liu et al., 2016).

### Histone Modifiers

Post-translational modification of histones also plays a key role in the regulation of chromatin dynamics. Transcriptionally active chromatins usually contain trimethylated histone H3K4 and highly acetylated histone H3 and H4. In contrast, transcriptionally silent chromatins are enriched in the methylation of lysine 9 and/or 27 of histone H3 (Hebbes and Thorne, 1988; Jenuwein and Allis, 2001; Fischle et al., 2003). Histone methylation is catalyzed by three distinct protein families; the SET domain-containing protein family, the non-SET domain proteins Dot1/Dot1L, and the PRMT1 family. In contrast to histone acetyl/ deacetyltransferases and based on early phylogenetic analysis, it was concluded that the SET domain-containing methyltransferases evolved in the eukaryotic lineage and the bacterial SET domain was the result of horizontal gene transfer from a eukaryotic host (Stephens et al., 1998; Iyer et al., 2003). However, a recent phylogenetic study using an expanded collection of prokaryotic genomes showed that the SET domain is found in free-living bacteria as well as in pathogenic bacteria. Interestingly, these enzymes are involved in the synthesis of secondary metabolites, such as antibiotics in bacteria (Iyer et al., 2011; Alvarez-Venegas, 2014). Thus, the SET domain is also an ancient catalytic domain. The SET-domain proteins are grouped into seven families (Ng et al., 2007) and are members of different complexes with broad functions. For example, polycomb group proteins (PcG) that act as chromatin-based transcriptional repressors, generally form two multimeric complexes, the polycomb repressive complexes 1 (PRC1) and PRC2. The histone methyltransferase Enhancer of Zeste [E(z)], which is the catalytic subunit of PRC2, catalyzes the trimethylation of histone H3 lysine 27 (H3K27me3) via its SET domain (Goodrich et al., 1997; Cao et al., 2002; Czermin et al., 2002). Arabidopsis consists of three H3K27me3 HMTs, CURLY LEAF (CLF), SWINGER (SWN), and MEDEA (MEA). The loss of function mutation of *CLF* and *SWN* that act, at least in part, redundantly leads to development of embryo- or callus-like structures in Arabidopsis (Goodrich et al., 1997; Grossniklaus et al., 1998; Chanvivattana et al., 2004). The prior positioning of H3K27me3 by the PRC2 complex is normally required for the recruitment of PRC1 and subsequent monoubiquitylation of histone H2A on lysine 119 (H2AK119ub1). However, PRC2 recruitment through PRC1-dependent H2A119ub1 has also been reported (Landeira et al., 2010; Blackledge et al., 2014). In contrast to PcG, the TRithoraX Group (trxG) proteins activate transcription by catalyzing methylation of histone H3 on lysine 4 (H3K4) via their SET domain. PcG and trxG proteins are essential in establishment and maintenance of cell identity and organ development in higher eukaryotes through permanent/dynamic transcriptional regulation of developmentally important genes (Alvarez-Venegas, 2010; Schuettengruber et al., 2017). Thus, they play a substantial role in morphological complexity. Phylogenetic analysis of the SET-domain proteins suggests that four families of the SET-domain proteins were present before the divergence of plants, metazoans, and fungi and later highly expanded and diverged in each kingdom mostly due to large-scale duplication (Zhang and Ma, 2012).

Histone demethylases are classified into two distinct families, the KDM1/LSD1 and JmjC domain-containing proteins. The catalytic domain of KDM1 genes is the AOD domain. The AOD domain is found in prokaryotes suggesting that prokaryotes are the provenance of eukaryotic KMD1-type HDMs. The eubacterial *Cupin* genes are likely the ancestor of all JmjC domain-containing proteins. Whole-genome duplication was likely the major driving force for the expansion and diversification of JmjC domain-containing proteins in complex multicellular eukaryotes (Qian et al., 2015). In contrast to eubacterial proteins that contain only the JmjC domain, most of the eukaryotic proteins contain complex architectural domains (Zhou and Ma, 2008; Qian et al., 2015).

Histone acetyltransferases and deacetylases both contain ancient catalytic domains, and members of the GCN5-related N-acetyltransferase (GNAT) superfamily and the histone deacetylase superfamily are found in all kingdoms of life. However, these enzymes were greatly expanded and diversified in multicellular eukaryotes (Leipe and Landsman, 1997; Gregoretti et al., 2004; Boycheva et al., 2014; Marinov and Lynch, 2016). HATs are grouped into two classes according to their intracellular localization, i.e., into A-type and B-type. B-type HATs are localized in the cytoplasm and catalyze acetylation of free histones. However, A-type HATs are localized in the nucleus and catalyze acetylation of the nucleosome core histones. In Arabidopsis, A-type HATs are classified into four groups based on their sequence and structural similarities (Eberharter et al., 1996; Pandey et al., 2002): (1) Gcn5-related N-acetyltransferases (GNATs), (2) The MYST-related HATs, (3) cAMP-responsive element-binding protein (CBP), and (4) TATA-binding protein associated factor (TAFII250). The HDACs are also classified into four groups: (1) Reduced Potassium Dependency 3 (RDP3), (2) Histone DeAcetylase 1 (HDA1), (3) Silent Information Regulator 2 (SIR2), and (4) Histone Deacetylase 2 (HD2) (Shen et al., 2015).

### Chromatin Remodelers

Transcription-relevant chromatin remodeling ATPases are classified into four distinct families (SWI/SNF, ISWI/SNF2L, CHD/Mi-2, and INO80/SWR1) that are functionally and genetically non-redundant based on their structure. The catalytic/ATPase domain of remodelers consists of two covalently linked RecA-like lobes. Chromatin remodeling complexes hydrolyze ATP and convert the chemical energy resulting from hydrolysis into mechanical motion, including sliding of the nucleosomes along the DNA, disassembling the nucleosome and exchanging histone variants (Flaus et al., 2006; Bannister and Kouzarides, 2011; Zhou et al., 2016). Phylogenetic studies have suggested that eukaryotic chromatin remodeling ATPases have likely evolved from the ancestral Snf2-like proteins in bacteria after the innovation of chromatin-binding domains in early eukaryotes (Flaus et al., 2006; Koster et al., 2015). The Arabidopsis orthologs of yeast SWI2/SNF2 are BRM, SYD, CHR12/MINU1, and CHR23/MINU2. Structurally, BRM is the closest ortholog to yeast SWI2/SNF2. It contains a helicase/SANT-associated (HAS) domain upstream of ATPase that is a binding platform for nuclear actin-related proteins (Szerlong et al., 2008) and a C-terminal bromodomain, which is capable of binding to acetylated lysine (Dhalluin et al., 1999; Jacobson et al., 2000). In *A. thaliana*, SWI2/SNF2 proteins assemble into different large complexes and control various activities such as plant growth and development (Sarnowska et al., 2016). The ISWI complexes were initially isolated from *D. melanogaster*. In *A. thaliana*, CHROMATIN REMODELING11 (CHR11) and CHR17 are orthologs of ISWI in *D. melanogaster*. They contain an ATPase domain at their N-terminus and HAND, SANT, and SLIDE domains at their C- terminus. AtISWI proteins, which are functionally redundant, form different complexes with the AtDDT (DNA-binding homeobox and different transcription factors)-domain proteins and control multiple developmental processes (Li et al., 2014). Proteins from the CHD/Mi-2 family contain two tandemly arranged chromodomains at the N-terminus that are able to interact with methylated histones and/or DNA. The CHD/Mi-2 family evolved soon after the onset of the eukaryotic lineage and further expanded in higher eukaryotes (Hargreaves and Crabtree, 2011; Gentry and Hennig, 2014; Koster et al., 2015). *Saccharomyces cerevisiae*, *A. thaliana*, and humans consist of one, four, and nine *CHD* genes, respectively (Koster et al., 2015). CHD remodelers positively or negatively control transcription and are also involved in mRNA processing (Murawska and Brehm, 2011; Hu et al., 2014). The chromatin-remodeling complexes of the INO80 group are INO80 and SWR1 in yeast. A single *INO80* and *SWR1/PIE1* (*PHOTOPERIOD INDEPENDENT EARLY FLOWERING 1*) are present in Arabidopsis. The INO80/SWR1 complexes similarly, to other chromatin-remodeling complexes work as transcriptional regulators. In addition, they are implicated in the DNA-repair system and are required for DNA recombination (Noh and Amasino, 2003; Fritsch et al., 2004; Gerhold and Gasser, 2014).

## Symbiosis and Multicellularity

It is well documented that mitochondria and chloroplasts of eukaryotic cells, which are descended from α-proteobacteria-like and cyanobacteria-like prokaryotes, respectively, arose through endosymbiosis (Weeden, 1981; Gray et al., 1999). Thus, endosymbiosis played a crucial role in the evolution of cellular complexity. Multicellular organisms harbor a vast diversity of microbes, comprising fungi, bacteria, protists, and viruses, collectively called microbiota (Almario et al., 2017; Durán et al., 2018). Molecular clock estimates of fungal phylogeny suggest that Ascomycota, Basidiomycota, and Glomales, which are major taxonomic groups of terrestrial fungi, were present around 600 myr ago (Redecker et al., 2000) and fossilized spores and fungal hyphae that are very similar to extant arbuscular mycorrhizal fungi (AMF) with the age of 460–480 myr support molecular estimates (Selosse and Le Tacon, 1998; Redecker et al., 2000; Heckman et al., 2001). Considering that early land plants colonized poorly developed soils and did not have true roots, the establishment of AMF symbiosis supplying nutrients, water, and enhancing tolerance to biotic and abiotic stresses was a key event in the terrestrialization process (Redecker et al., 2000; Heckman et al., 2001; Rausch et al., 2001; Kenrick and Strullu-Derrien, 2014; Almario et al., 2017; Xue et al., 2018). In addition to fungi, bacterial micribiota are a substantial part of diverse assemblages of symbiotic microorganisms and are critical for plant survival (Durán et al., 2018). Surprisingly, bacterial symbiosis is required for cell division and morphogenesis in *Ulva mutabilis*, which is a green macroalgae and an important primary producer in coastal ecosystems (Wichard, 2015). Taken together, these lines of evidences suggest that symbiosis played an important role in the transition from water to land and the evolution of multicellularity. Organism-associated microbes had a great impact on phenotypic extension and host evolution. In evolutionary studies, considering the host and its associated microbiota as a biological entity, the holobiont could be key for a better understanding of the evolution of multicellular organisms (Shropshire and Bordenstein, 2016; Almario et al., 2017; Hassani et al., 2018; Haag, 2018).

## Conclusion

In early eukaryotes, due to an increase of genome size, high density packaging of the DNA molecules into the confined space of the nucleus and simultaneous evolution of novel factors controlling the accessibility of DNA was a necessity to ensure all DNA-based processes, including transcriptional regulation. Increased genome size together with higher available energy per gene likely led to the evolution of chromatin structure and chromatin-modifying factors in early eukaryotes (Flaus et al., 2006; Lane and Martin, 2010; Koster et al., 2015; Garg and Martin, 2016; Martin and Sousa, 2016). Although, the origins of catalytic subunits of chromatin remodelers and modifiers can be traced back in prokaryotes, these catalytic subunits and their interacting partners continuously expanded and highly diversified and were finally coopted, while prokaryotes lack chromatin-remodeling and –modifying complexes. The innovation of these complexes was a key prerequisite step in the evolutionary trajectory of complex multicellular eukaryotes. Both symbiotic microbiota and epigenetics are critical for adaptation to environmental conditions, plant survival, and their evolution. However, our knowledge concerning how diversification and expansion of chromatin-related factors and recruitment of symbiotic microbiota led to the complexity of living organisms is low. In addition, the functional links between symbiotic microbiota and epigenetics is largely unknown. In future work, a combination of approaches in ecophysiology, plant-microbe interaction, phylogenomics, molecular biology, systems biology, cell biology, and biochemistry studies on a wide range of unicellular and multicelluar organisms will shed more light on the interrelationship of chromatin-related factors and microbiota community structure and their contribution to the evolution of complex multicellular organisms and the holobiont.

## Author Contributions

MH wrote the manuscript. CK and MB critically revised and approved the manuscript for publication.

## Conflict of Interest Statement

The authors declare that the research was conducted in the absence of any commercial or financial relationships that could be construed as a potential conflict of interest.
